# The Dynamic Response of Nitrogen Transformation to the Dissolved Oxygen Variations in the Simulated Biofilm Reactor

**DOI:** 10.3390/ijerph18073633

**Published:** 2021-03-31

**Authors:** Qianqian Lu, Nannan Zhang, Chen Chen, Miao Zhang, Dehua Zhao, Shuqing An

**Affiliations:** School of Life Science, Nanjing University, Nanjing 210023, China; dg1930057@smail.nju.edu.cn (Q.L.); 18487262425@163.com (N.Z.); snowcchen@163.com (C.C.); miaozhangmz@163.com (M.Z.); anshq@nju.edu.cn (S.A.)

**Keywords:** simulated biofilm reactor, stable state, nitrogen cycle, dissolved oxygen, gene activity

## Abstract

Lab-scale simulated biofilm reactors, including aerated reactors disturbed by short-term aeration interruption (AE-D) and non-aerated reactors disturbed by short-term aeration (AN-D), were established to study the stable-state (SS) formation and recovery after disturbance for nitrogen transformation in terms of dissolved oxygen (DO), removal efficiency (RE) of NH_4_^+^-N and NO_3_^−^-N and activity of key nitrogen-cycle functional genes *amo*A and *nir*S (RNA level abundance, per ball). SS formation and recovery of DO were completed in 0.56–7.75 h after transition between aeration (Ae) and aeration stop (As). In terms of pollutant REs, new temporary SS formation required 30.7–52.3 h after Ae and As interruptions, and seven-day Ae/As interruptions required 5.0% to 115.5% longer recovery times compared to one-day interruptions in AE-D and AN-D systems. According to *amo*A activity, 60.8 h were required in AE-D systems to establish new temporary SS after As interruptions, and RNA *amo*A copies (copy number/microliter) decreased 88.5%, while 287.2 h were required in AN-D systems, and RNA *amo*A copies (copy number/microliter) increased 36.4 times. For *nir*S activity, 75.2–85.8 h were required to establish new SSs after Ae and As interruptions. The results suggested that new temporary SS formation and recovery in terms of DO, pollutant REs and *amo*A and *nir*S gene activities could be modelled by logistic functions. It is concluded that temporary SS formation and recovery after Ae and As interruptions occurred at asynchronous rates in terms of DO, pollutant REs and *amo*A and *nir*S gene activities. Because of DO fluctuations, the quantitative relationship between gene activity and pollutant RE remains a challenge.

## 1. Introduction

Influent water stabilization to wastewater treatment systems (WWTSs) in terms of pollutant concentration and composition has been widely implemented as a strategy to stabilize the effluent water quality and realize sustainable operation [1,2]. Currently, the resilience of a WWTS as well as the response of a WWTS to dramatic influent variations has drawn increasing attention in both academia and industry, because of the rapid socioeconomic development and global climatic change [2,3]. Two kinds of strategies have been widely adopted to increase WWTS resilience, i.e., increasing the ability of a WWTS to handle shock loads and decreasing the load variation extent by building buffering facilities before pumping to the WWTS occurs. Despite high construction and maintenance costs, buffering facilities such as automated control and storm tanks are still the most common measures to increase WWTS resilience [4]. In a simulated biofilm reactor (SBR), there generally exists a notable linear correlation between the influent and effluent water quality levels, and thus influent stabilization is an important condition for achieving water quality discharge standards [1,5]. Therefore, in large-scale integrated BR systems for wastewater treatment, stabilization, detention or aeration ponds are designed as the front end of sequential facilities to buffer any possible shock loads in terms of the quantity or composition [1,3,6].

Nitrogen is an important indicator of eutrophication, which is an important factor affecting water environment and public health [7,8]. The removal of nitrogen from wastewater has always been a concentrated research direction in the field of wastewater treatment. Biofilm reactors including constructed wetlands are a way to remove excess nitrogen from water, and are widely used in wastewater treatment plants and natural water environment. DO is an important factor affecting the N cycle reaction, including nitrification (NH_4_^+^-N→NO_3_^−^-N ) and denitrification (NO_3_^−^-N →N_2_) [9,10,11,12]. Ammoxidation (NH_4_^+^-N→NO_2_^−^-N) and nitrite reduction (NO_2_^−^-N→NO) are rate-limiting steps for nitrification and denitrification, with amoA and nirS+nirK as marker genes, respectively [13,14]. Nitrification and denitrification are important processes in N removal, although the N removal process is very complex. Changes in DO could affect the rate of nutrient removal such as N [9,10,11,12]. However, there is limited research on the mechanism of microbial response to DO changes, especially the dynamic gene activity during the stable state formation and recovery.

Regarding the dissolved oxygen (DO) in BRs, the conditions are quite different. For the removal of nitrogen (N) from eutrophic water in BRs, creating spatial or temporal alternatives of aerobic and anaerobic states is an important strategy because nitrification followed by denitrification is the dominant N removal pathway in most BR systems [9,10,11,12]. In BRs, several technologies have been implemented to establish temporal alternatives of aerobic and anaerobic states, for example, tide-flow and sequential-batch influent modes resulting not only in fluctuations in the pollutant concentration but also in periodic aerobic and anaerobic state transformations [15], with intermittent aeration causing periodic and frequent aerobic and anaerobic state transformations [16]. Although much effort has been gone into the identification of the optimal operation strategies for tide-flow and sequential-batch influent BRs [17,18], as well as the optimal artificial aeration modes [10], there exists a research gap in BRs, i.e., the stable-state (SS) formation process under altered DO conditions and the DO recovery process to its original level [19].

Numerous studies have demonstrated the stimulation of the nitrification and denitrification functional gene abundance levels in the aerobic and anaerobic states, respectively [20,21,22]. However, the formation of N functional gene abundance, measured from the extracted DNA of biofilms, as well as the stable microbiological community in BRs, require a relatively long period [22,23]. Moreover, the microbial community generally remains relatively stable once formation occurs [24]. Instead of the functional gene abundance, gene activity probably showed closer relationship with the functional enzyme activity or the N transformation rate [22,25]. Therefore, the widely used marker gene abundance, such as nirK, nirS, and nosZ genes being used as the marker genes to study the denitrification process [13], is probably not suitable for the identification of microbial mechanisms in studying the response of nitrogen transformation to frequent DO variations [26]. Currently, limited studies have focused on the long- and short-term dynamic responses of BRs to dramatic DO variations [19,24]. To our knowledge, no attempts have been conducted to identify the dynamic effect of frequent DO variations on the N-cycle functional gene activity, the dynamics of new SS formation and recovery process of the gene activity, or its relationship with the N-cycle, which has a special significance for the identification of microbial mechanisms in studying the response of N transformation to the currently widely used intermittent aeration and variable inflow modes in BRs.

The objective of this study was to investigate the dynamic process of the responses of the aerobic and anaerobic SSs (SS-AE and SS-AN, respectively) in BRs to DO fluctuations by intermittent air charging, which is important for the optimization of the DO supply to improve the N removal efficiency (RE) in BRs as well as to identify the underlying microbial mechanism. The influent water is simulated wastewater with typical constituents of COD, NH_4_^+^-N, NO_3_^−^-N, and micro-element. The response process included the SS-AE and SS-AN formation, disturbance and recovery under DO fluctuations in terms of (1) DO consumption, (2) NH_4_^+^-N and NO_3_^−^-N Res, and (3) activity of key N-cycle function genes *amo*A and *nir*S, which are the rate-limiting genes in the nitrification and denitrification process, the most important pathways of nitrogen transformation in BRs treating wastewater despite the complex microbial nitrogen-cycling network [14,27].

## 2. Materials and Methods

### 2.1. The Simulated BR System

**Figure 1:**
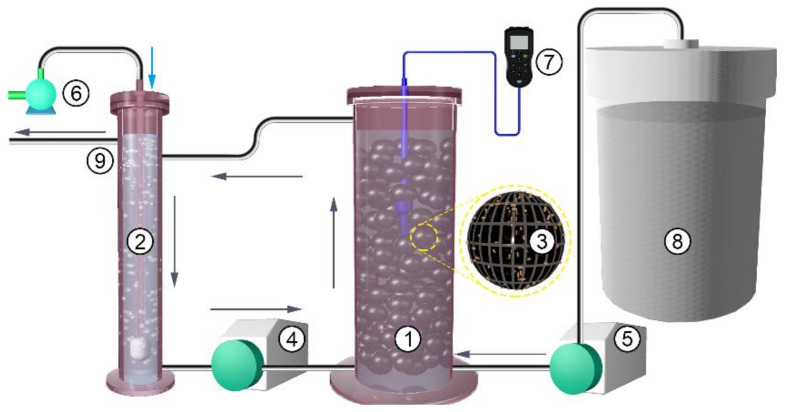
Schematic diagram of the simulated biofilm reactor (BR) system. **1**. The main reactor; **2**. the regulative reactor; **3**. the substrate (the biological balls); **4**. the peristaltic pump driving the internal circulation; **5**. the peristaltic pump controlling the influent rate; **6**. the air pump for aeration; **7**. the dissolved oxygen (DO) meter; **8**. the wastewater tank; **9**. the export of water.

An indoor lab-scale SBR system was set up at Nanjing University, Nanjing city, China, in 2018. The system was composed of two incubators: a large incubator with dimensions of 0.04^2^ × π × 0.50 m^3^, acting as the main reactor, and a small incubator with dimensions of 0.02^2^ × π × 0.50 m^3^, acting as the regulative reactor (Figure 1). The main and regulative incubators were connected by a peristaltic pump (a Rainin Dynamax model RP-1, Hampton, NH, USA) and a return silicone tube (ShenChen, Baoding, China). Water was regularly pumped into the main reactor at a rate of 50 mL/min (i.e., one circle was completed in approximately 40 min) to fully mix the water in the system. Another peristaltic pump (ShenChen, model LabM3, Baoding, China) was used at an influent feed rate of 2 mL/min, thereby establishing a hydraulic retention time of 0.69 days. Instead of the widely used natural substrates such as gravel and ceramsite, artificial substrates consisting of biological balls (Yafeng, Shijiazhuang China) were implemented, which were made of high-density porous polyethylene (HDPP), the material density is 1.27 g/cm^3^, and specific surface area of the biological ball is 390 m^2^/m^3^. A DO detector (RDO-202, Chemins, China) and aerator header (LB808, China) were installed at the middle of the main reactor and at the bottom of the regulative reactor, respectively.

Our SBR system was slightly different from the current widely used BRs. First, in addition to the main reactor, the SBR included an internal circulation design with a relatively high circulation water rate to completely mix the water in the SBR system, thus establishing relatively uniform water conditions and substrate biofilms in different layers, which can improve the representativeness of the sampled water and substrate for microbiological parameter measurements. Second, instead of a natural substrate, biological balls were adopted to not only improve the representativeness of the sampled microbes due to the high ball uniformity but also to increase the extraction efficiency of RNA and functional enzymes from the attached substrate biofilms because the balls are easier to grind than the natural substrate. At the same time, the use of biological balls as well as to decrease the disturbances from iron sorption and desorption from the substrates to achieve of our objective (i.e., evaluate the effect of DO variations on the N transformation in BRs by microbes).

### 2.2. Experimental Design and Operation Conditions

**Figure 2:**
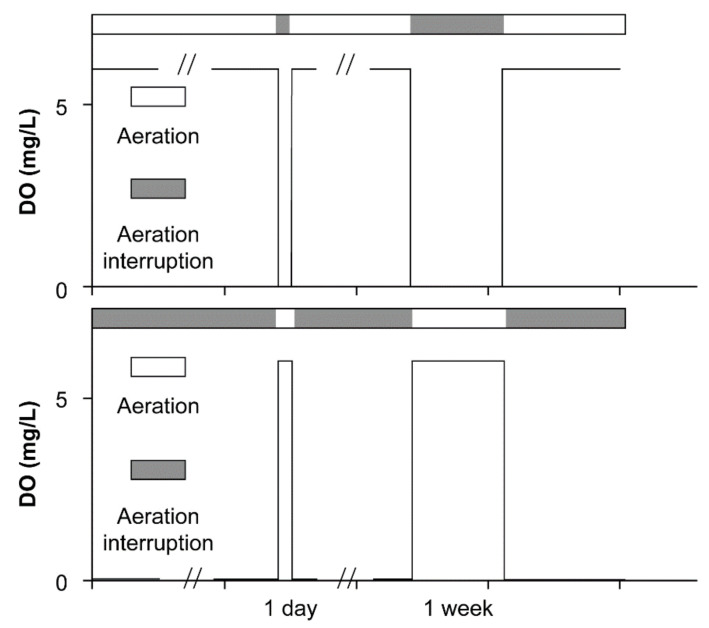
The designed conceptual modes for the transition between the aerobic and anaerobic states of the simulated BR.

Two types of reactors were designed, i.e., aerobic steady-state reactors with aeration interruptions (AE-D) and anaerobic steady-state reactors with intermittent aeration (AN-D). For the AE-D reactors, after aerobic SS formation, evaluated by the standard whereby both the effluent NO_3_^−^-N and NH_4_^+^-N concentrations should vary no more than 10% in three measurements in one week, one-day aerobic interruptions were performed, and the reactors were then returned to the aerobic state until the original aerobic steady state was recovered, evaluated by the same aforementioned standard. Similarly, one-week aerobic disturbances were carried out, after which the reactors were again returned to the aerobic steady state. For the AN-D reactors, after anaerobic SS formation, one-day and one-week aerobic disturbances were also conducted (Figure 2). In this study, the DO in the anaerobic and aerobic states approaches zero and is higher than 5.0 mg/L, respectively. The excessive aeration in the regulative reactor, the internal water circulation between the large and small incubators, and the relatively high chemical oxygen demand (COD) concentration resulted in the rapid transition between the aerobic and anaerobic states.

As the aerobic and anaerobic SS formation processes in BRs and their recovery from disturbance, previous studies were based on the recovery time to the baseline and a mathematical model of the microbial biomass/community or effluent water quality [3,23]. In contrast, a simple methodology was developed to quantify the disturbance and recovery processes of the aerobic and anaerobic SSs, with the time as the independent parameter and the DO, the N-NO_3_^−^/N-NH_4_^+^ RE or the functional gene activity related to nitrogen transformation as the dependent parameters. A logistic function was adopted [28]:

(1) $$y=\frac{c}{\left(1+e^{a+b*x}\right)}+d$$

where x is the sampling day, y is the DO, N-NO_3_^−^/N-NH_4_^+^ RE or functional gene activity related to nitrogen transformation, c and d are the potential minimum and maximum values, respectively, of the DO, N-NO_3_^−^/N-NH_4_^+^ RE or functional gene activity, and a and b are regression coefficients.

After the establishment of the above logistic model, the curvature change rate of the model was calculated (i.e., the k’ value according to [28]), and subsequently, the transition points were determined to acquire the time of SS formation or recovery from disturbance [28]. The inflection point of the curve corresponds to the transition point at which the N-NO_3_^−^/N-NH_4_^+^ RE or functional gene activity entered the stable and slow growth period.

The activated sludge was obtained from the nearby Tiebei wastewater treatment plant (Nanjing, China) for treating municipal sewage. The aerobic and anaerobic activated sludge were respectively collected from the aeration tank, packed into clean polyethylene plastic drums, and transported back to the laboratory as soon as possible at low temperature. Then, 100 mL supernatant was extracted and added to the system to accelerate biofilm formation. The continuous inflow mode was sustained by the peristaltic pump. The influent water quality parameters were an NH_4_^+^-N concentration of 60 mg/L (Ammonium sulfate, Aladdin, Shanghai China), an NO_3_^−^-N concentration of 30 mg/L (Potassium nitrate, Aladdin, Shanghai, China), and a COD concentration of 450 mg/L (methanal, Aladdin, Shanghai, China). Macronutrients of Ca, P, K, and S and micronutrients were supplied with a 1:3 Hoagland standard solution [29]. The water temperature in the research period was automatically recorded with an automatic temperature recorder, HOBO U12-012 (Onset, Bourne, MA, USA). During the experimental period, the water temperature fluctuated between 22.9 and 25.5 °C, with an average of 24.3 °C.

### 2.3. Sampling and Measurement

The effluent water was sampled for the measurement of the NH_4_^+^-N and NO_3_^−^-N concentrations every 8 h to 7 days using a Hach DR/3900 spectrophotometer and standard operating procedures. The NO_3_^−^-N concentration was determined by chromium reduction method and colorimetry with a spectrophotometer. The concentration of NH_4_^+^-N was determined by the salicylic acid method and colorimetry using a spectrophotometer. The sampling interval (SI) varied with the stability of the effluent NH_4_^+^-N and NO_3_^−^-N REs in the reactor: during the initial periods (i.e., longer than two months after the operation of the reactor) and the disturbance periods of intermittent aeration, the SIs lasted 2–4 days; the SIs lasted 8 h during the initial three days of the recovery period after the aeration disturbance (aeration interruption or commencement) and then lasted 2–7 days.

After water sampling, three biological balls were tested from each reactor system for biofilm characteristic analysis. Since ammonia oxidation and nitrite conversion into nitric oxide are the rate-limiting steps of nitrification and denitrification, respectively, *amo*A and *nir*S genes, respectively, were measured [27].

The RNA abundance of the *amo*A and *nir*S genes was determined using real-time quantitative PCR (qPCR) (termed as RNA level amoA and nirS genes). One biological ball was sampled from each reactor and placed in 50-mL sterile tubes. RNA was extracted using the TRIzol method. Next, RNA was inverted to cDNA using the RT Kit HiScript II Q RT Supermix for qPCR (Vazyme Biotech, Nanjing, China), and primers amo598f(5′GAATATGTTCGCCTGATTG3′)/amo718r(5′CAAAGTACCACCATACGCAG3′) [30] and nirScd3aF(5′GTSAACGTSAAGGARACSGG3′)/nirSR3cd(5′GASTTCGGRTGSGTCTTGA3′) [31] were used. The qPCR assays were performed on an ABI StepOnePlus qPCR detection system (ABI, USA) using the ChamQ SYBR qPCR master mix (high-ROX premixed) by Vazyme Biotech. Each 20-μL reaction mixture contained 10 μL 2×ChamQ SYBR qPCR master mix, 2 μL cDNA template, 0.4 μL each of the relevant forward and reverse primers (10 μM), and ddH_2_O. The amoA gene analysis parameters were 10 min at 95 °C, 15 s at 95 °C, 45 s at 56 °C, and 30 s at 72 °C. The nirS gene analysis parameters were 10 min at 95 °C, 15 s at 95 °C, 30 s at 58 °C, and 30 s at 72 °C. The plasmids of the target genes were manufactured by the Majorbio Bio-pharm Technology Company (Shanghai, China), and these plasmids were diluted 10 times into six solutions to generate standard curves. The standard curves for the target genes had R^2^ values between 0.999 and 1, and the amplification efficiency ranged from 92.3 to 107.2%.

The statistical analysis of difference significance was conducted by Student’s *t*-test. 

## 3. Results and Discussion

### 3.1. The Transformation between the SSs in Terms of the DO

**Figure 3:**
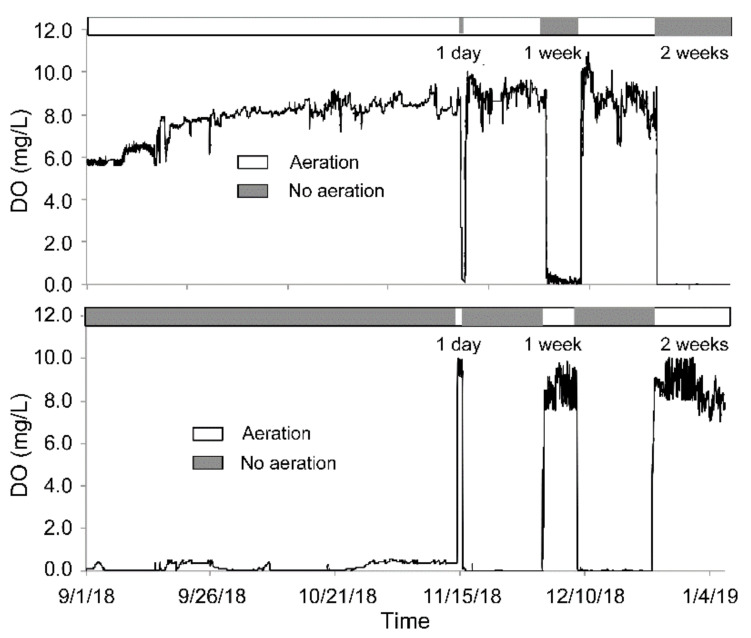
The measured dissolved oxygen concentration dynamics in the aerated reactors disturbed by short-term aeration interruption (AE-D) (above) and non-aerated reactors disturbed by short-term aeration (AN-D) (below) reactors.

As designed, the transitions between Ae and As resulted in rapid transformations between the aerobic and anaerobic SS in DO (SS-AE_DO_ and SS-AN_DO_, respectively) values in both the AE-D and AN-D reactors (Figure 2 and Figure 3). For the AE-D reactors, 7.75, 5.25, and 4.36 h were required for the transformation from SS-AE_DO_ to SS-AN_DO_ after aeration was interrupted for 1, 7, and 14 days, respectively, while 0.76, 0.56, and 0.56 h, respectively, were required for the transition of SS-AN_DO_ to the original SS-AE_DO_ after aeration was restarted after aeration had been interrupted for 1, 7, and 14 days, respectively. At the attained SS-AE_DO_, the DO concentration fluctuated between 5.60 and 10.96 mg/L with an average of 8.35 mg/L, while at the attained SS-AN_DO_, the DO concentration ranged from 0.00 to 0.77 mg/L with an average of 0.047 mg/L.

For the AN-D reactors, aeration induced the transformation from SS-AN_DO_ to SS-AE_DO_ over 0.70, 0.69, and 0.72 h after the 1-, 7-, and 14-day interruptions, respectively, while the As conditions resulted in the recovery from the attained SS-AE_DO_ to the original SS-AN_DO_ after 2.19, 2.42, and 3.78 h after the 1-, 7-, and 14-day interruptions, respectively. At the attained SS-AN_DO_, the DO concentration fluctuated between 0.00 and 0.38 mg/L, with an average of 0.12 mg/L, while at the attained SS-AE_DO_, the DO concentration ranged from 7.01 to 10.04 mg/L, with an average of 8.57 mg/L.

For both the AE-D and AN-D reactors, the transformation from the anaerobic SS to the aerobic SS was quicker than the reverse transformation, which was probably related to the over-aeration of the reactor systems. Compared with the AE-D reactors, the AN-D reactors required significantly shorter times for the transformation from SS-AE_DO_ to SS-AN_DO_ after As was introduced (*p* = 0.03), which was obtained from Student’s t-test. There was no significant difference between the AE-D and AN-D reactors in the time required for the transformation from SS-AN_DO_ to SS-AE_DO_ after aeration was restarted (*p* = 0.32). A significant relationship was observed between the time required to attain SS-AN_DO_ from SS-AE_DO_ after As was introduced and the number of aeration days in the past 30 days before As occurred (*p* = 0.01), which suggested that the high-SS-AN_DO_ systems easily recovered the original SS-AN_DO_ after a short-term aeration interruption. However, SS-AE_DO_ formation from the original SS-AN_DO_ after aeration was restarted was not significantly correlated with the number of aeration days in the past 30 days before re-aeration (*p* = 0.55). In addition, both SS-AN_DO_ and SS-AE_DO_ were established more quickly than was mentioned in previous reports [19,24], which was probably related to the rapid internal water circulation between the main and regulative reactors, as well as the excessive aeration conditions in this study.

### 3.2. The Transformation between the SSs in Terms of the NH_4_^+^-N and NO_3_^−^-N REs

#### 3.2.1. The Formation of a New Temporary SS

**Figure 4:**
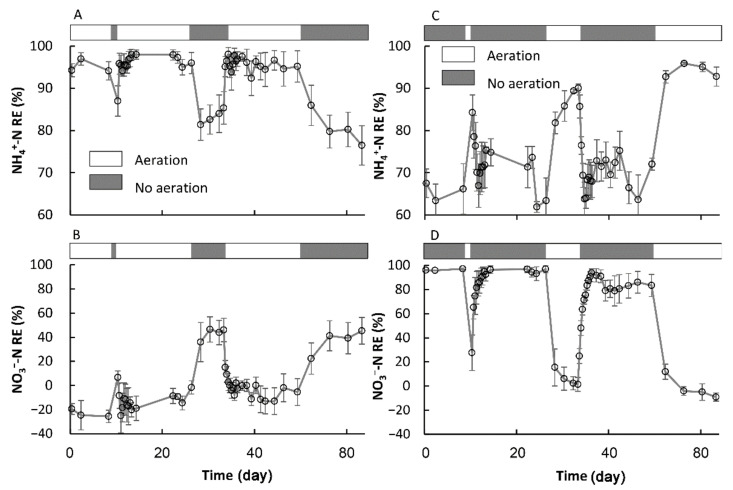
The fluctuations in the NH_4_^+^-N and NO_3_^−^-N removal efficiencies with the transformation from aeration to no aeration in the AE-D (**A**,**B**) and AN-D reactors (**C**,**D**). (sample number: 44).

**Figure 5:**
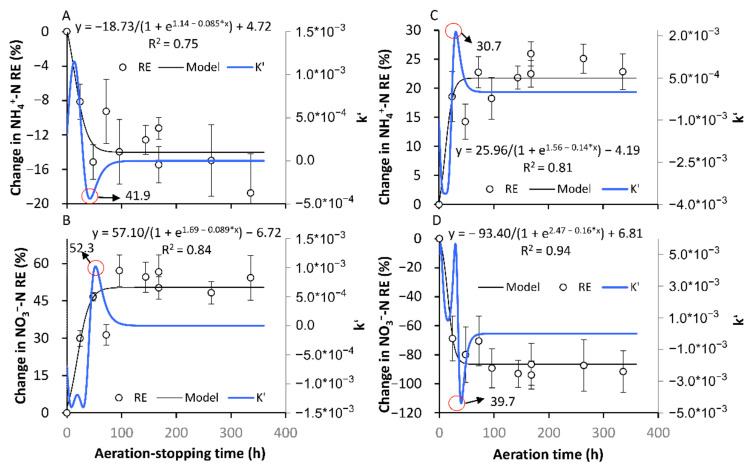
The formation processes of the new stable states in the removal efficiencies of NH_4_^+^-N and NO_3_^−^-N after the transformation from aeration to no aeration. (**A**,**B**) show the formation of SS-AN_NH4+_ and SS-AN_NO3−_, respectively, in the AE-D reactors; (**C**,**D**) show the formation of SS-AE_NH4+_ and SS-AE_NO3−_, respectively, in the AN-D reactors. The red circle is the position of the inflection point, and the number indicated by the arrow is the corresponding time of the inflection point. (sample number: 10).

As expected, the transformation between Ae and As resulted in substantial fluctuations in the NO_3_^−^-N and NH_4_^+^-N REs in both the AE-D and AN-D reactors (Figure 4 and Figure 5). For the AE-D reactors, the NH_4_^+^-N and NO_3_^−^-N REs were 76.5–98.1% and −25.6 to 46.5%, respectively, while the AN-D reactors exhibited NH_4_^+^-N and NO_3_^−^-N REs of 61.9–95.9% and −9.2 to 97.3%, respectively. Despite the large fluctuations, both the AE-D and AN-D reactors generally attained a relatively high NH_4_^+^-N RE, which was probably related to the multiple NH_4_^+^-N removal pathways from the systems, including aerobic nitrification and other pathways [14,24], while the NO_3_^−^-N RE fluctuated greatly. During most As periods, the relatively high NO_3_^−^-N RE probably resulted from the sufficient carbon source supply due to the inhibition of organic matter degradation under anaerobic conditions, thus leading to the relatively high denitrification level [32]. In contrast, the relatively low NO_3_^−^-N RE in most Ae periods was probably determined by the production of NO_3_^−^-N from NH_4_^+^-N nitrification and denitrification inhibition [14].

For both the AE-D and AN-D reactors, the short-term interruptions of the Ae/As conditions resulted in relatively large fluctuations in the NH_4_^+^-N and NO_3_^−^-N REs (Figure 5). For the AE-D systems, the NH_4_^+^-N and NO_3_^−^-N REs gradually changed with As over time, and 41.9 and 52.3 h were required to reach the new anaerobic SS in NH_4_^+^-N (SS-AN_NH4+_) and NO_3_^−^-N (SS-AN_NO3−_) REs, respectively, after As was introduced. For the AN-D systems, 30.7 and 39.7 h were required after Ae occurred to reach the new aerobic SS in NH_4_^+^-N (SS-AE_NH4+_) and NO_3_^−^-N (SS-AE_NO3−_) REs, respectively. The attainment of both the new SS-AN_NH4+_ from the original SS-AE_NH4+_ and the new SS-AN_NO3−_ from the original SS-AE_NO3−_ in the AE-D systems required slightly longer times than were needed to reach the new SS-AE_NH4+_ and SS-AE_NO3−_ in the AN-D systems, which was probably related to the higher rate of DO replenishment in the aeration-restarting period than that of DO consumption in the no aeration period (Figure 2). Excluding the difference between the DO replenishment and consumption rates, reaching the aerobic SS NH_4_^+^-N and NO_3_^−^-N REs was similar to that of the anaerobic SS REs. Attaining the new SS-AN_NO3−_ in the AE-D systems and the new SS-AE_NO3−_ in the AN-D systems required slightly longer times than were needed to reach the new SS-AN_NH4+_ in the AE-D systems and the new SS-AE_NH4+_ in the AN-D systems, which can be explained by the generation of NO_3_^−^-N from NH_4_^+^-N nitrification; thus, the SS formation of NO_3_^−^-N must occur after the SS formation of NH_4_^+^-N. However, the substantially higher NH_4_^+^-N RE as well as the substantially lower NO_3_^−^-N RE in the AE-D systems than those in the AN-D systems suggested that both the SS-AN_NO3−_ and SS-AN_NH4+_ conditions were temporary SSs that were different from the systems established under long-term aerobic or anaerobic conditions [24].

#### 3.2.2. The Recovery of the Original SS in Terms of the NH_4_^+^-N and NO_3_^−^-N REs

**Figure 6:**
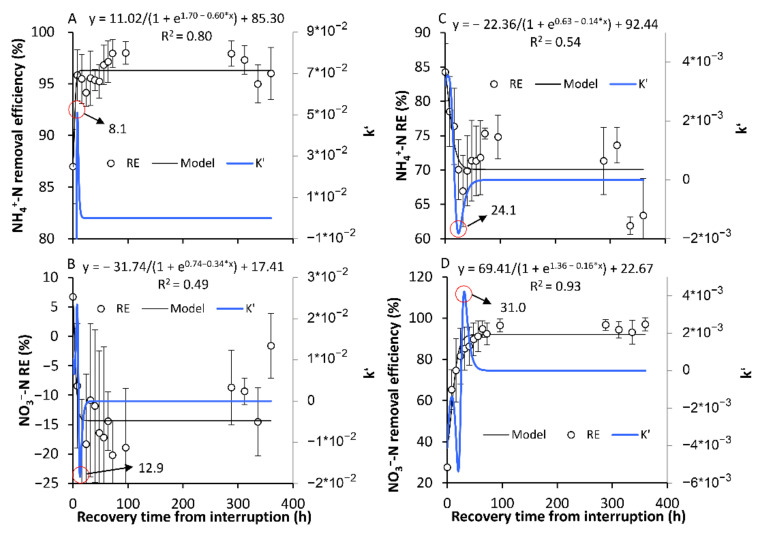
The recovery back to the original stable state (SS) of the removal efficiency of NH_4_^+^-N and NO_3_^−^-N after the one-day aeration or no-aeration interruption. (**A**,**B**) show the recoveries of the original SS of the removal efficiency of NH_4_^+^-N and NO_3_^−^-N, respectively, in the AE-D reactors; (**C**,**D**) show the establishment of the original SS of the removal efficiency of NH_4_^+^-N and NO_3_^−^-N, respectively, in the AN-D reactors. The red circle is the position of the inflection point, and the number indicated by the arrow is the corresponding time of the inflection point. (sample number: 15).

**Figure 7:**
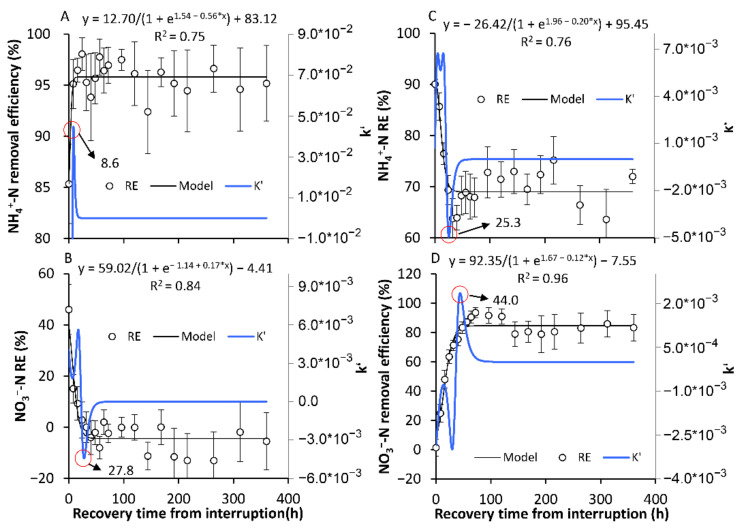
The recovery back to the original stable state (SS) of the removal efficiency of NH_4_^+^-N and NO_3_^−^-N after the seven-day aeration or no aeration interruption. (**A**,**B**) show the recovery of the original SS of the removal efficiency of NH_4_^+^-N and NO_3_^−^-N, respectively, in the AE-D reactors; (**C**,**D**) show the recovery of the original SS of the removal efficiency of NH_4_^+^-N and NO_3_^−^-N, respectively, in the AN-D reactors. The red circle is the position of the inflection point, and the number indicated by the arrow is the corresponding time of the inflection point. (sample number: 19)

After the one-day or one-week Ae/AS interruption, the NH_4_^+^-N and NO_3_^−^-N REs gradually recovered to the SS before the interruptions (Figure 6 and Figure 7). For the AE-D reactors, the recovery times were 8.1 and 12.9 h for NH_4_^+^-N and NO_3_^−^-N, respectively, after the one-day Ae interruption, while for the AN-D reactors, the recovery times were 24.1 and 31.0 h for NH_4_^+^-N and NO_3_^−^-N, respectively, after the one-day As interruption. Compared with the one-day Ae/As interruptions, a longer recovery time was needed after the seven-day interruptions, with the time increasing 5.0% to 115.5%. Therefore, certain conclusions can be drawn. (1) The longer Ae/As interruptions require a longer recovery time to again reach the original SS NH_4_^+^-N and NO_3_^−^-N REs. (2) Compared with the AE-D reactors, the AN-D reactors generally require a longer recovery time after the Ae/As interruptions. (3) To again attain the original RE, a longer time is required for NO_3_^−^-N than for NH_4_^+^-N after interruption.

There are multiple pathways for the removal of NH_4_^+^-N and NO_3_^−^-N from the reactors whereby NH_4_^+^-N and NO_3_^−^-N can continuously form and mutually be transformed by nitrification and assimilative denitrification [14]. Therefore, even though sometimes the NH_4_^+^-N and NO_3_^−^-N REs remain relatively stable, the abundance and activity of the microorganisms in biofilms are constantly evolving [23]. The above results suggested that for the relatively stable systems formed in a long-term stable environment, the transition from Ae to As conditions can result in temporary new SS NH_4_^+^-N and NO_3_^−^-N REs within a short time (30.7–52.3 h). However, the temporary new SSs are different from the original SSs before the transformation formed under long-term Ae/As conditions. Our results also revealed that the formation of new SSs and recovery of the NH_4_^+^-N or NO_3_^−^-N REs from the interruption occurred significantly slower than those in the DO.

The relatively short times for SS formation and recovery of the NH_4_^+^-N and NO_3_^−^-N REs from the interruption, which were considerably shorter than the generation times of nitrification and denitrification bacteria [33], suggested that the RE fluctuations were mainly induced by variations in the nitrogen-related microbial activity instead of the abundance. The similar times for the establishment of aerobic and anaerobic SS NH_4_^+^-N or NO_3_^−^-N REs suggested that the response speed of the related microbial activity was probably similar between the DO stimulation and inhibition periods. In addition, another issue should be noted. The pollutant RE was calculated by the variation between the influent and effluent concentrations, which was not the instantaneous real removal rate of the system when the pollutant concentration in the system varied due to the Ae/As interruption. Excluding the time spent on the gradual replenishment or consumption of the varied pollutant concentrations before and after the attainment of the new SS, the response time of the active nitrogen-cycle related microbes to the Ae/As interruption should also be shorter than the measured formation or recovery time of the SS NH_4_^+^-N and NO_3_^−^-N REs.

### 3.3. The Transformation between SSs in Terms of the Key Nitrification and Denitrification Genes

#### 3.3.1. The Formation of a New SS in Terms of the amoA and nirS Activities

**Figure 8:**
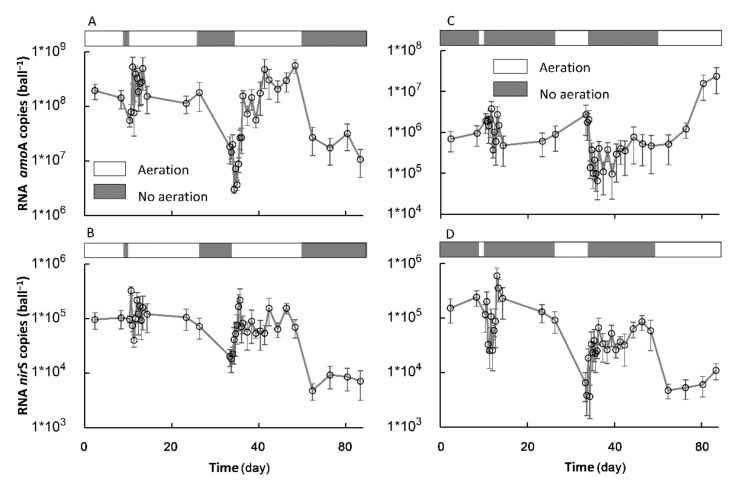
The fluctuations in the abundance of nitrogen-cycle functional genes *amo*A and *nir*S at the RNA level with the transformation between aeration and no aeration in the AE-D (**A**,**B**, respectively) and AN-D reactors (**C**,**D**, respectively) (sample number: 38).

The Ae/As interruptions resulted in large fluctuations in the activity of the nitrification and denitrification genes in terms of the RNA level of the *amo*A and *nir*S copies, respectively (Figure 8). During the whole experimental period, the *amo*A copies ranged from 2.99 × 10^6^ to 5.58 × 10^8^ and from 6.55 × 10^4^ to 2.35 × 10^7^, with averages of 1.61 × 10^8^ and 1.92 × 10^6^, respectively, in each ball for the AE-D and AN-D systems, respectively, while the *nir*S copies ranged from 4.76 × 10^3^ to 3.248 × 10^5^ and from 3.63 × 10^3^ to 5.91 × 10^5^, with averages of 9.04 × 10^4^ and 8.18 × 10^4^, respectively, in each ball for the AE-D and AN-D systems, respectively. The results suggested that both the *amo*A and *nir*S activities were sensitive to the Ae/As interruptions. This result is consistent with previous studies reporting that oxygen was one of the important factors determining the nitrification and denitrification activities as well as the abundance and activity of *amo*A and *nir*S [34,35,36].

**Figure 9:**
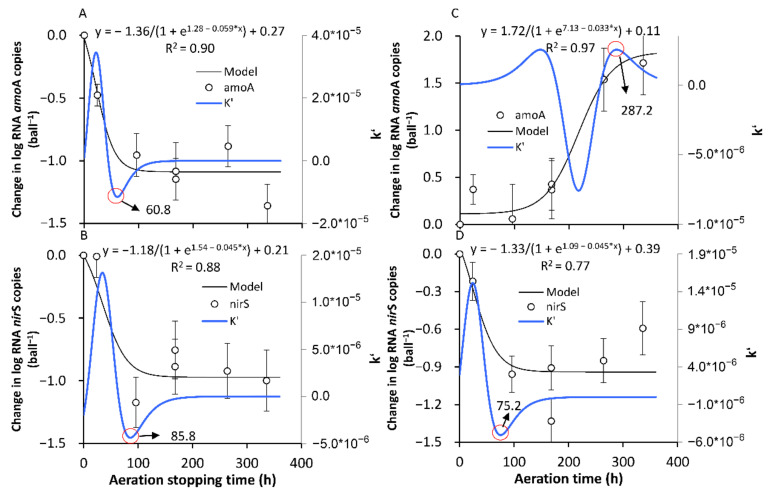
The formation processes of the new stable states of the RNA level abundance of nitrogen-cycle functional genes *amo*A and *nir*S after the transformation between aeration and no aeration. (**A**,**B**) show the formation of SS-AN*_amo_*_A_ and SS-AN*_nir_*_S_, respectively, in the AE-D reactors; (**C**,**D**) show the formation of SS-AE*_amo_*_A_ and SS-AE*_nir_*_S_, respectively, in the AN-D reactors. The red circle is the position of the inflection point, and the number indicated by the arrow is the corresponding time of the inflection point. (sample number: 7).

The Ae interruptions in the AE-D reactors gradually decreased the *amo*A activity in the RNA level *amo*A copies, while the As interruptions in the AN-D reactors gradually increased the *amo*A activity (Figure 9). In the AE-D reactors, 60.8 h were required to attain the new SS-AN of the *amo*A activity (SS-AN*_amo_*_A_) from the original SS-AE of the *amo*A activity (SS-AE*_amo_*_A_) after the As interruptions, and the RNA *amo*A copies in each ball decreased 88.5%, while 287.2 h were required in the AN-D reactors to attain the new SS-AE*_amo_*_A_ from the original SS-AN*_amo_*_A_ level after the Ae interruptions, and the RNA *amo*A copies in each ball increased 36.4 times. However, the RNA *amo*A copies at both the attained SS-AE*_amo_*_A_ and SS-AN*_amo_*_A_ in the AE-D reactors were much more abundant than those at both the attained SS-AE*_amo_*_A_ and SS-AN*_amo_*_A_ in the AN-D reactors, which suggested that both the new SS-AE*_amo_*_A_ and SS-AN*_amo_*_A_ levels were temporary instead of actual long-term SSs (Figure 8). The results suggested that the high *amo*A activity of SS-AE*_amo_*_A_ would be quickly lost due to anaerobic disturbance and that a new temporary SS-AN*_amo_*_A_ with a relatively low *amo*A activity would be attained in a short time (i.e., 60.8 h under our experimental conditions). In contrast, a relatively long time is required to attain a new temporary SS-AE*_amo_*_A_ with a high *amo*A activity due to aeration from the original SS-AN*_amo_*_A_ with a low *amo*A activity, i.e., 287.2 h under our experimental conditions.

The establishment of a new temporary SS-AN*_amo_*_A_ in the AE-D reactors and a new SS-AE*_amo_*_A_ in the AN-D reactors took longer than that of a new SS-AN_NH4+_ in the AE-D reactors and a new SS-AE_NH4+_ in the AN-D reactors (Figure 5A,C, respectively). The unsynchronization between the NH_4_^+^-N RE and the limiting functional gene activity of nitrification *amo*A is probably related to the multiple pathways of NH_4_^+^-N removal in addition to nitrification [37,38].

In contrast to *amo*A, the RNA *nir*S copies in both the AE-D and AN-D reactors decreased slightly after the Ae/As interruptions (Figure 9). In the AE-D reactors, 85.8 h were required for the establishment of a new SS-AN of the *nir*S activity (SS-AN*_nir_*_S_) from the original SS-AE of the *nir*S activity (SS-AE*_nir_*_S_) after the Ae interruptions, which was similar to the time required for the establishment of a new SS-AE*_nir_*_S_ after the As interruptions in the AN-D reactors (75.2 h). There was no significant difference in *nir*S activity between the SS-AE*_nir_*_S_ conditions in the AE-D reactors and the SS-AN*_nir_*_S_ conditions in the AN-D reactors (*p* = 0.55), with averages of 9.22 × 10^5^ and 6.07 × 10^5^ RNA *nir*S copies at the attained SS-AE*_nir_*_S_ in the AE-D reactors and at the attained SS-AN*_nir_*_S_ in the AN-D reactors, respectively. The slight difference was probably the result of the synthetic influence of multiple factors, including the nitrogen substrate (the total N, NH_4_^+^-N, and NO_3_^−^-N), available phosphorus (P), organic matter, DO concentration, and pH [27,35]. However, the SS-AE*_nir_*_S_ denitrifiers were probably mainly distributed in the biofilm interlayers under aerobic conditions in the AE-D reactors, while the SS-AN*_nir_*_S_ denitrifiers probably occurred in both the inter- and outer-layers of the biofilms in the AN-D reactors [27,39]. The *nir*S activity decrease after the Ae interruptions in the AE-D reactors probably resulted from the NO_3_^−^-N limitation due to the NO_3_^−^-N reduction by nitrification (Figure 5B,D) [27], while the *nir*S activity decrease after the As interruptions in the AN-D reactors probably occurred due to the carbon source limitation resulting from the more rapid degradation under aerobic conditions than that under anaerobic conditions and resulting from oxygen inhibition under aerobic conditions [27,40,41].

The interruptions in the AE-D and AN-D reactors had different influences on the NO_3_^−^-N RE and *nir*S activity: the establishment of new SS-AE*_nir_*_S_ and SS-AN*_nir_*_S_ required more time than that of the new SS-AE_NO3−_ and SS-AN_NO3−_; the Ae interruptions in AE-D reactors resulted in an increase in the NO_3_^−^-N RE and a decrease in the *nir*S activity (Figure 5B,D). The different responses between the NO_3_^−^-N RE and *nir*S activity suggested that it was probably inappropriate to characterize the NO_3_^−^-N RE with the *nir*S activity; although, the *nir*S and *nir*K abundance and activity levels have been widely used as gene markers for denitrifiers [14,42], which is probably related to the existence of several NO_3_^−^-N removal pathways in addition to canonical denitrification, such as partial denitrification and aerobic denitrification [43].

#### 3.3.2. The Recovery of the Original SS in Terms of the amoA and nirS Activities

**Figure 10:**
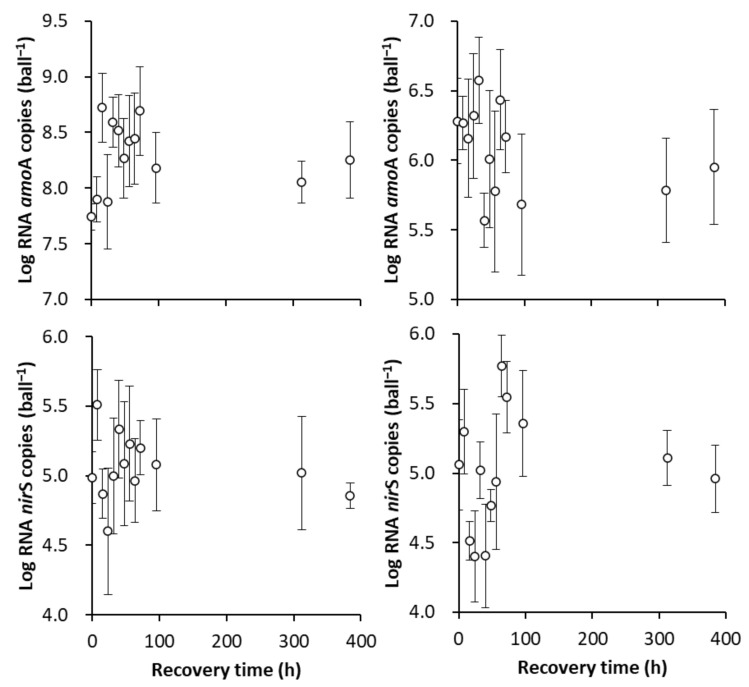
The recovery of the original stable state (SS) of the RNA level abundance of nitrogen-cycle functional genes *amo*A and *nir*S after the one-day aeration or no aeration interruption. (**A**,**B**) show the recovery of the original SS of the RNA level abundances of *amo*A and *nir*S, respectively, in the AE-D reactors; (**C**,**D**) show the recovery of the original SS of the RNA level abundances of *amo*A and *nir*S, respectively, in the AN-D reactors. (sample number: 13).

**Figure 11:**
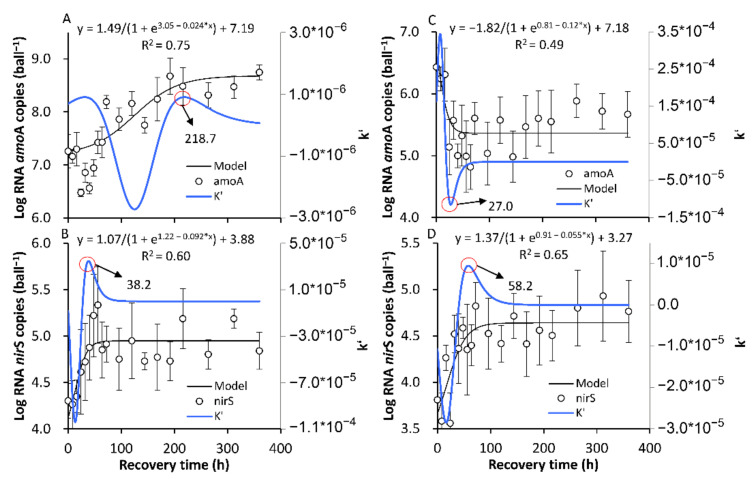
The recovery of the original stable state (SS) of the RNA level abundance of nitrogen-cycle functional genes *amo*A and *nir*S after the seven-day aeration or no aeration interruption. (**A**,**B**) show the recovery of the original SS of the RNA level abundances of *amo*A and *nir*S, respectively, in the AE-D reactors; (**C**,**D**) show the recovery of the original SS of the RNA level abundances of *amo*A and *nir*S, respectively, in the AN-D reactors (sample number: 19).

Despite the significant variations in *amo*A and *nir*S activities along with the Ae/As interruptions, the limited variation extent resulting from the one-day interruption resulted in a relatively low correlation between the number of RNA *amo*A and *nir*S copies and the recovery time based on the determination coefficient (R^2^) after the one-day interruption (Figure 10). Therefore, only the recovery after the seven-day interruption was investigated (Figure 11). It took 218.7 and 27.0 h to recover the original *amo*A activity after the seven-day Ae/As interruptions in the AE-D and AN-D reactors, respectively (Figure 11A,C, respectively). These results suggested that it was relatively difficult (i.e., a long time was required) to recover the original SS-AE*_amo_*_A_ level with a high *amo*A activity in the AE-D reactors, while it was relatively easy (i.e., a short time was required) to recover the original SS-AN*_amo_*_A_ with a low *amo*A activity in the AN-D reactors. It took 38.2 and 58.2 d to recover the original SS-AE*_nir_*_S_ and SS-AN*_nir_*_S_ levels with a high *nir*S activity after the seven-day interruptions in both the AE-D and AN-D reactors, respectively (Figure 11B,D, respectively), which suggested that recovering the *nir*S activity was relatively easy after both the Ae and As interruptions.

Based on the establishment of a new SS after the Ae/As interruption and the recovery of the original SS after the interruption of the *amo*A and *nir*S activities (Figure 9 and Figure 11), it can be concluded that it is relatively difficult for both the formation and recovery of a high *amo*A activity under aerobic conditions, while it is relatively easy (i.e., a short time is needed) to lose the high *amo*A activity. For the *nir*S activity, it is relatively easy to both lose and recover the high *nir*S activity after the transformation from Ae to As. Therefore, long-term stable aerobic conditions are important for maintaining a high *amo*A activity in biofilms, while a high *nir*S activity in biofilms can be attained under both stable aerobic and anaerobic conditions. Although DO promotion of *amo*A and DO inhibition of *nir*S in both abundance and activity have been widely identified [27], the disturbance and recovery dynamics caused by DO variations still represent a research gap, which is important for the mechanistic examination of the frequent DO fluctuations in many BRs with management strategies such as temporal intermittent oxygen supplementation [10].

The recovery of the high *amo*A activity after Ae interruption substantially lagged behind that of the high NH_4_^+^-N RE (Figure 6, Figure 7 and Figure 11). This result suggested that there probably existed other NH_4_^+^-N removal pathways that were more rapidly recovered by the restart of aeration, which need to be identified in future experiments using enzyme inhibitors or the more comprehensive analysis of nitrogen-cycle gene activity dynamics [26,44]. In fact, although multiple NH_4_^+^-N removal pathways, nitrifiers, and influencing factors have been identified [27], few studies have been carried out on the response dynamics of the nitrogen-cycle gene activity and NH_4_^+^-N removal pathways under DO variation conditions, which may probably be useful for the development of new strategies with higher nitrogen REs.

## 4. Conclusions

The processes of new temporary SS formation and recovery to the original level with the transformation between Ae and As were modelled using logistic functions in terms of the DO, NH_4_^+^-N, and NO_3_^−^-N REs and the RNA abundance of *amo*A and *nir*S.

In regard to the DO, the transformation from SS-AE_DO_ to SS-AN_DO_ required 2.19–7.75 h after the As interruption, which was significantly faster than the reverse transformation, requiring 0.56–0.76 h after the Ae interruption. The formation or recovery of SS-AN_DO_ required a longer time than that of SS-AE_DO_ after the transformation from Ae to As. There was a significant correlation between the time required to reach SS-AN_DO_ from SS-AE_DO_ after the As interruption and the number of aeration days in the past 30 days for both systems.

In terms of the pollutant RE, the establishment of SS-AN_NH4+_ and SS-AN_NO3−,_ after the As interruptions in the AE-D reactors, required 41.9 and 52.3 h, respectively. At the same time, 30.7 and 39.7 h were required for the establishment of SS-AE_NH4+_ and SS-AE_NO3−_, respectively, after the Ae interruptions in the AN-D reactors. After the one-day Ae/As interruptions, the recovery time ranged from 8.1 to 31.0 h, while after the seven-day Ae/As interruptions, 5.0% to 115.5% longer recovery times were required. The longer Ae/As interruptions resulted in a longer recovery time. The AN-D reactors generally required a longer recovery time than the AE-D reactors after the Ae/As disruptions. A longer time was required for NO_3_^−^-N than for NH_4_^+^-N to recover after the Ae/As disruptions.

Regarding the *amo*A activity, 60.8 h were required in the AE-D reactors to reach the new temporary SS-AN*_amo_*_A_ after the As interruptions, and the RNA *amo*A copies increased 88.5%, while 287.2 h were required in the AN-D reactors to reach the new SS-AE*_amo_*_A_ after the Ae interruptions, and the RNA *amo*A copies increased 36.4 times. In terms of the *nir*S activity, 75.2–85.8 h were needed for the establishment of SS-AE*_nir_*_S_ or SS-AN*_nir_*_S_ after the Ae or As interruptions. After the 7-day Ae/As interruptions, 218.7 and 27.0 d were required to recover the original SS-AE*_amo_*_A_ and SS-AN*_amo_*_A_ in the AE-D and AN-D reactors, respectively, while 38.2–58.2 h were needed to recover the *nir*S activity.

Therefore, the temporary SS formation and recovery after the Ae and As interruptions in terms of the DO, nitrogen removal efficiency (NH_4_^+^-N and NO_3_^−^-N) and gene activities (*amo*A and *nir*S) required tens of minutes to several hours, several to tens of hours, and tens to hundreds of hours, respectively. The asynchronous variation in the DO, NH_4_^+^-N, and NO_3_^−^-N REs and activity of key nitrogen-cycle genes after the Ae and As interruptions suggests that their quantitative relationship needs to be further investigated. In addition to nitrification and denitrification, there are many ways in the nitrogen cycle, so more research is needed to explore the formation of stable state and the recovery mechanism of disturbances.

## Data Availability

The data presented in this study are available on request from the corresponding author. The data are not publicly available due to the subsequent research needs.

## Nomenclature

Ae	Aeration
AE-D	Aeration disturbed by a short-term aeration interruption
As	Aeration stop
AN-D	No aeration disturbed by short-term aeration
COD	Chemical oxygen demand
SBR	Simulated biofilm reactor
DO	Dissolved oxygen
NH_4_^+^-N	Ammoniacal nitrogen
NO_3_^−^-N	Nitrate nitrogen
RE	Removal efficiency
SS	Stable state
SS-AE	Aerobic stable state
SS-AE*_amo_*_A_	Aerobic stable state of the *amo*A activity
SS-AE_DO_	Aerobic stable state of the DO
SS-AE_NH4+_	Aerobic stable state of the NH_4_^+^-N removal efficiency
SS-AE*_nir_*_S_	Aerobic stable state of the *nir*S activity
SS-AE_NO3−_	Aerobic stable state of the NO_3_^−^-N removal efficiency
SS-AN	Anaerobic stable state
SS-AN*_amo_*_A_	Anaerobic stable state of the *amo*A activity
SS-AN_DO_	Anaerobic stable state of the DO
SS-AN_NH4+_	Anaerobic stable state of the NH_4_^+^-N removal efficiency
SS-AN*_nir_*_S_	Anaerobic stable state of the *nir*S activity
SS-AN_NO3−_	Anaerobic stable state of the NO_3_^−^-N removal efficiency
SS-AN_DO_	Anaerobic stable state of the DO
WWTS	Wastewater treatment system
